# Linkage disequilibrium patterns, population structure and diversity analysis in a worldwide durum wheat collection including Argentinian genotypes

**DOI:** 10.1186/s12864-021-07519-z

**Published:** 2021-04-05

**Authors:** Pablo Federico Roncallo, Adelina Olga Larsen, Ana Laura Achilli, Carolina Saint Pierre, Cristian Andrés Gallo, Susanne Dreisigacker, Viviana Echenique

**Affiliations:** 1 Centro de Recursos Naturales Renovables de la Zona Semiárida (CERZOS), Departamento de Agronomía, Universidad Nacional del Sur (UNS)-CONICET, Bahía Blanca, Argentina; 2 CEI Barrow, Instituto Nacional de Tecnología Agropecuaria (INTA), Tres Arroyos, Buenos Aires Argentina; 3 International Maize and Wheat Improvement Center (CIMMYT), El Batán, Edo. de México Mexico

**Keywords:** Durum, Linkage disequilibrium, Population structure, SNP, Diversity, Rare alleles

## Abstract

**Background:**

Durum wheat (*Triticum turgidum* L. ssp. *durum* Desf. Husn) is the main staple crop used to make pasta products worldwide. Under the current climate change scenarios, genetic variability within a crop plays a crucial role in the successful release of new varieties with high yields and wide crop adaptation. In this study we evaluated a durum wheat collection consisting of 197 genotypes that mainly comprised a historical set of Argentinian germplasm but also included worldwide accessions.

**Results:**

We assessed the genetic diversity, population structure and linkage disequilibrium (LD) patterns in this collection using a 35 K SNP array. The level of polymorphism was considered, taking account of the frequent and rare allelic variants. A total of 1547 polymorphic SNPs was located within annotated genes. Genetic diversity in the germplasm collection increased slightly from 1915 to 2010. However, a reduction in genetic diversity using SNPs with rare allelic variants was observed after 1979. However, larger numbers of rare private alleles were observed in the 2000–2009 period, indicating that a high reservoir of rare alleles is still present among the recent germplasm in a very low frequency. The percentage of pairwise loci in LD in the durum genome was low (13.4%) in our collection. Overall LD and the high (*r*^*2*^ > 0.7) or complete (*r*^*2*^ = 1) LD presented different patterns in the chromosomes. The LD increased over three main breeding periods (1915–1979, 1980–1999 and 2000–2020).

**Conclusions:**

Our results suggest that breeding and selection have impacted differently on the A and B genomes, particularly on chromosome 6A and 2A. The collection was structured in five sub-populations and modern Argentinian accessions (cluster Q4) which were clearly differentiated. Our study contributes to the understanding of the complexity of Argentinian durum wheat germplasm and to derive future breeding strategies enhancing the use of genetic diversity in a more efficient and targeted way.

**Supplementary Information:**

The online version contains supplementary material available at 10.1186/s12864-021-07519-z.

## Background

Durum wheat (*Triticum turgidum* L. ssp. *durum* Desf. Husn) is one of the most important food crops in the world [1] with a worldwide production of about 36 millon tons [2]. It was derived from wild Emmer wheat (*T. turgidum* ssp*. dicoccoides*, 2n = 4X = 28, AABB) in a two-step domestication process that took place in the Fertile Crescent (10,000 BP) and now it is cultivated globally [3]. The main producing areas of durum wheat are in the Mediterranean Basin, North America and India, Canada and Turkey being the main producer countries of this cereal, followed by Argelia, Italy and India [4]. Historically it has been used as the main source for making different products, mainly flat and leavened bread, couscous, burghul and frekeh in the West Asia, and the North and East Africa region and to elaborate pasta in Western Europe, as well as in North America and worldwide [5]. It has been suggested that durum wheat was the first type of wheat sown in the Spanish colonies in South America in 1527 [6]. In Argentina, the widespread cultivation of durum started with the introduction of European or Asian landraces, followed by the beginning of wheat breeding during the first two decades of the XXth century. The incorporation of the semi-dwarf genes (*Rht*) during the green revolution occurred during the 70’s. The older cultivars, typically conformed by high and less productive plants, were progressively replaced before the beginning of the 80’s and all the durum wheat varieties cultivated in Argentina today are semi-dwarf [7]. Argentina annually cultivates the largest durum wheat area in South America (53,480 ha in 2019/20) (http://datosestimaciones.magyp.gob.ar/) mainly in the southeast of Buenos Aires province, but also in the north-center of the country in Tucumán province and minor areas in San Luis and Córdoba. Durum wheat grains are mainly used for dry pasta production, one of the main staple foods in Argentina, with a consumption of 8.54 kg per capita p.a. and occupying the 7th worldwide position of production and consumption [8].

The understanding of genetic diversity available in this crop provides breeders with important knowledge to 1) properly design future strategies in plant breeding, 2) assist in germplasm collection management, and 3) conserve diversity in the national genebanks. To evaluate the genetic diversity in durum wheat, different wheat germplasm collections have been established and genetically characterized using DNA markers by several research institutions [9–17]. Genetic diversity in modern cultivars is usually decreased due to bottleneck events during domestication [18] and strong selection in breeding [13, 19]. However, some authors [17] have found a low or null decay in diversity from landraces to modern cultivars, although they observed an effect of breeding on the linkage disequilibrium (LD) patterns and allele’s frequency. Efforts in recovering genetic diversity and to capture beneficial alleles for specific traits have been made by exploring the genetic variability available in landraces [20–24] and wild relatives [25–27].

Single Nucleotide Polymorphisms (SNPs) are the most common type of polymorphism in genomes [28]. The use of array technologies developed to capture variants in SNP markers in wheat has become a cost-effective and more efficient way to assess diverse genetic resources [29]. Several wheat SNP arrays, such as the 9 K or 15 K Infinium BeadChip [30] and the 90 K iSelect SNP Array [31] from Illumina (https://www.illumina.com), or the 820 K Wheat HD genotyping Array [32], the 35 K Axiom Wheat Breeder’s Array [33] and the Wheat 660 K Array [34] from Affymetrix (www.affymetrix.com), are available and have been widely used during recent years. Furthermore, Next-generation sequencing (NGS) based approaches, such as Genotyping by Sequencing (GBS) [35], or DArtSeq [36], and other emerging technologies are powerful tools for SNP discovery. The sequencing of hexaploid (bread) and tetraploid (wild emmer and durum) wheat genomes [37–39] has anchored the molecular markers to their physical positions.

The study of LD can be defined as the nonrandom association of alleles at different loci due to genetic linkage, as well as artificial selection, drift, bottlenecks and other genetic forces [40]. Previous studies have addressed this issue in durum wheat [10, 41–43]. However, the analysis of LD patterns in a germplasm collection including Argentinian durum wheat by using an SNP array has not yet been performed. The study of LD could help to understand the effect of selection pressure exerted over the national germplasm that occurred during the breeding processes. An initial genetic characterization of a subset of the durum wheat collection used in this study was performed with AFLP and a low number of KASP™ SNPs markers [14]. For the present study our goals were to i) assess the genetic diversity in a collection of 197 durum wheat accessions ii) study the population structure in our germplasm collection to establish the main genetic relationships between the Argentinian durum wheat and other foreign germplasm, iii) estimate LD patterns considering the variation in the genome, population structure and the time of release of evaluated genotypes.

## Results

### Distribution and physical location of polymorphic SNPs

From all the SNP results, 7431 SNPs were high-quality polymorphic in the 197 durum wheat accessions (Additional file 1: Table S1a, b), of which 4854 (65.3%) SNPs showed and minor allele frequency (MAF) > 0.05, hereafter called high frequency (HF) SNPs and 2577 (34.7%) corresponded to ˋrare alleles´ SNP with an MAF < 0.05, subsequently called low frequency (LF) SNPs. A total of 7222 out of 7431 polymorphic SNPs could be aligned to the Svevo whole genome sequence assembly with an average inter-marker distance of 1.38 Mb, whereas the HF and LF SNPs showed average values of 2.1 Mb and 4.0 Mb, respectively. The SNP distribution in the durum wheat genome is shown in Table 1. The number of SNPs per chromosome ranged from 231 (4A) to 542 (1B) for HF SNPs whereas the LF SNPs varied from 70 (4B) to 337 (1B). The HF SNPs were better distributed than the LF SNPs. The B genome had a higher number of polymorphic SNPs, where 1B, 2B and 6B chromosomes showed higher representation. The annotation’s ID and function of genes containing SNPs were listed in Additional file 2: Tables S2a, b. A total of 1547 polymorphic SNPs was located within the annotated genes, out of which 595 corresponded to LF SNPs and 952 to HF SNPs. Out of these, 16 annotated genes carried three or more than three SNP markers, and in particular, two annotations (TRITD6Bv1G225150 and TRITD7Av1G001490) showed nine and six polymorphic SNPs, respectively (Additional file 2: Tables S2c, d).

**Table 1:** Genome distribution of SNP markers, genetic diversity and linkage disequilibrium indices

Chr	HF SNPs												LF SNPs						Total SNP
	N	Marker coverage (Mb)	MAF	Ho	He	LD (***r***^***2***^) ^**a**^	% LD ^**b**^	LD decay (Mb)	% ***r***^***2***^ < 0.1	ARG LD (***r***^***2***^) ^**c**^	SNPs on annotated genes ^**d**^	N (Filtered Subset) ^**e**^	N	Marker coverage (Mb)	MAF	Ho	He	SNPs on annotated genes ^**d**^	
1A	305	1.92	0.237	0.021	0.324	0.177	18.5	14.7	63.3	0.294	50	45	221	2.64	0.013	0.002	0.026	46	526
1B	542	1.26	0.264	0.020	0.350	0.162	27.0	19.1	60.5	0.272	118	79	337	2.02	0.012	0.002	0.023	106	879
2A	365	2.13	0.220	0.018	0.303	0.220	19.5	9.8	56.2	0.433	69	29	178	4.36	0.020	0.003	0.039	37	543
2B	427	1.85	0.240	0.021	0.328	0.151	21.8	14.2	61.6	0.284	84	56	251	3.13	0.017	0.002	0.034	68	678
3A	275	2.72	0.242	0.015	0.329	0.153	26.5	14.9	64.3	0.287	46	37	184	4.07	0.015	0.002	0.029	31	459
3B	288	2.91	0.281	0.020	0.361	0.157	19.2	9.8	63.3	0.274	62	49	271	3.09	0.016	0.002	0.031	71	559
4A	231	3.19	0.237	0.019	0.328	0.177	17.2	10.8	65.6	0.304	47	32	100	7.41	0.015	0.003	0.030	23	331
4B	246	1.90	0.253	0.017	0.347	0.192	19.4	14.9	63.0	0.300	58	41	70	9.76	0.014	0.002	0.028	8	316
5A	284	2.35	0.237	0.019	0.320	0.153	19.6	10.5	65.1	0.287	48	41	165	4.07	0.013	0.003	0.026	34	449
5B	344	2.04	0.261	0.017	0.345	0.155	19.3	14.4	62.5	0.288	66	51	161	4.36	0.016	0.003	0.031	46	505
6A	277	2.23	0.239	0.019	0.320	0.290	15.1	8.6	56.2	0.465	40	29	104	5.95	0.021	0.003	0.041	20	381
6B	413	1.69	0.241	0.019	0.327	0.153	15.9	9.5	65.8	0.268	97	47	188	3.65	0.016	0.002	0.030	45	601
7A	380	1.91	0.240	0.019	0.331	0.173	19.7	5.6	62.9	0.322	77	59	153	4.74	0.021	0.003	0.040	39	533
7B	362	2.00	0.239	0.019	0.335	0.137	21.4	8.7	66.6	0.288	90	57	100	7.28	0.023	0.003	0.044	21	462
A genome	2117	2.35	0.236	0.019	0.322	0.192	19.4	10.7	61.7	0.345	377	272	1105	4.747	0.017	0.002	0.033	230	3222
B genome	2622	1.95	0.254	0.019	0.342	0.158	20.6	12.9	62.7	0.278	575	380	1378	4.755	0.016	0.002	0.031	365	4000
Unmapped	115	.	0.254	0.022	0.34	0.116	.	.	.	.	.	23	94	.	0.018	0.003	0.035	.	209
Whole genome	**4854**	2.10	0.246	0.019	0.333	0.090	13.4	11.8	62.3	0.302	952	675	**2577**	4.01	0.016	0.002	0.031	595	**7431**

*HF* High frequency, *LF* low frequency, *N* number of SNPs, *MAF* minor allele frequency, *Ho* observed heterozygosity, *He* expected heterozygosity (Nei’s gene diversity), *LD* linkage disequilibrium

^a^ mean intra-chomosomal LD at *p* < 0.01

^b^ Percentage of pairwise SNPs in significant LD (*p* < 0.01)

^c^ Mean LD calculated considering only 85 Argentinian accessions

^d^ SNPs located into annotated genes in the Svevo genome assembly

^e^ Selected SNPs with intra-chromosomal distance > 1 Mb and MAF > 0.3

### Genetic diversity analysis

Genetic diversity was analyzed in all the chromosomes considering HF and LF SNPs separately. Nei’s gene diversity (*He*) considering HF SNPs was higher for the B genome, showing maximum values on the 3B and 1B chromosomes, while the A genome showed higher values of *He* for LF SNPs (rare allele) (Table 1). When the geographical origin or period of release were taken into account the private alleles (alleles that are found only in a single subgroup) were not observed among the HF SNPs (Table 2). However, the analysis of rare alleles detected 1102 and 1122 private alleles based on geographical origin and the period of the genotype’s breeding or release, respectively.

**Table 2:** Genetic diversity estimated in the whole collection and subgroups

Subgroup	N	4854 HF SNPs						2577 LF SNPs					
		%PL	*Na*	*I*	*Ho*	*He*	PA	%PL	*Na*	*I*	*Ho*	*He*	PA
**Origin** ^**a**^													
ARM	71	98.0	1.98	0.478	0.029	0.315	0	45.4	1.45	0.051	0.003	0.022	200
ART	14	83.8	1.84	0.416	0.016	0.273	0	19.6	1.20	0.056	0.002	0.031	50
CHI	26	80.6	1.81	0.390	0.008	0.257	0	27.7	1.28	0.057	0.001	0.028	303
CIM	10	66.3	1.66	0.348	0.003	0.231	0	9.1	1.09	0.032	0.001	0.019	1
FRA	22	92.4	1.92	0.462	0.024	0.306	0	24.6	1.25	0.061	0.003	0.032	86
ITM	16	81.4	1.81	0.423	0.008	0.282	0	12.3	1.12	0.041	0.002	0.023	18
ITT	17	91.7	1.92	0.457	0.020	0.301	0	48.3	1.48	0.131	0.005	0.070	416
USA	4	53.3	1.53	0.320	0.016	0.220	0	6.8	1.07	0.039	0.002	0.026	29
WAN	17	84.7	1.85	0.424	0.008	0.280	0	17.4	1.17	0.048	0.001	0.026	26
**Period**													
1915–1959	6	70.3	1.70	0.382	0.015	0.255	0	10.6	1.11	0.047	0.002	0.029	12
1960–1969	5	61.5	1.62	0.352	0.018	0.239	0	19.6	1.20	0.098	0.006	0.064	33
1970–1979	15	91.0	1.91	0.460	0.022	0.304	0	48.1	1.48	0.137	0.005	0.074	396
1980–1989	22	94.9	1.95	0.474	0.017	0.314	0	23.4	1.23	0.049	0.002	0.024	30
1990–1999	24	95.2	1.95	0.482	0.008	0.320	0	22.7	1.23	0.046	0.001	0.022	32
2000–2009	101	99.8	2.00	0.487	0.015	0.320	0	71.1	1.71	0.067	0.002	0.028	590
2010–2020	24	92.5	1.93	0.459	0.048	0.303	0	19.2	1.19	0.034	0.003	0.016	29
**DAPC**													
Q1	68	99.2	1.99	0.478	0.023	0.315	1	54.2	1.54	0.066	0.003	0.029	313
Q2	41	97.3	1.97	0.450	0.019	0.293	0	35.2	1.35	0.050	0.002	0.022	104
Q3	36	92.0	1.92	0.419	0.014	0.271	0	56.0	1.56	0.108	0.003	0.054	511
Q4	18	70.6	1.71	0.327	0.030	0.212	1	8.2	1.08	0.019	0.002	0.010	6
5	34	83.2	1.83	0.364	0.011	0.234	0	31.2	1.31	0.056	0.001	0.027	297
Total	197	100	2.00	0.503	0.019	0.333	–	100	2.00	0.078	0.002	0.031	–

*HF* high frequency, *LF* low frequency, *% PL* percentage of polymorphic loci, *Na* average number of alleles, *I* Shannon’s Information index, *Ho* observed heterozygosity, *He* Nei’s gene diversity or heterozygosity, *PA* number of private alleles

Q1 to Q5 are the sub-population inferred by DAPC

^a^ *ARM* Accessions are coded as: modern Argentinian, *ART* traditional Argentinian, *CHI* Chile, *CIM* CIMMYT, *FRA* France, *ITM* modern Italian, *ITT* traditional Italian, *USA* United States, *WAN* West Asia/ North Africa region. Accessions from Argentina and Italy were divided into two groups according to the breeding period or year of release (until: ʽtraditional,ʼ and after 1985: ʽmodernʼ)

The highest genetic diversity indices (*I*, *He*, *Ho*, *Na*, %PL) calculated using HF SNPs were observed in the modern Argentinian accessions (ARM), followed by the French (FRA) and traditional Italian ones (ITT), whereas the lowest indices were observed in the genotypes from the USA, CIMMYT and Chile (Additional file 3: Figure S1a, b). However, when the indices and the number of private alleles (PA) were based on LF SNPs, the ITT constituted the most diverse subgroup. All 17 ITT accessions carried rare PAs and 416 LF SNP variants that were exclusive of this subgroup (37.7% of total) giving an average of 24.5 PA by accessions (Additional file 4: Table S3a). The Chilean (303 PA) and modern Argentinian (200 PA) subgroups also captured a high number of rare SNP variants. The PCoA via distance matrix with data standardization of the Nei genetic distance evidenced that modern Argentinian genotypes are genetically related to WANA region accessions. On the other hand, Chilean accessions were closely related to CIMMYT germplasm (Additional file 5: Table S4a).

Diversity indices calculated according to the period of the genotype’s breeding or release were also analyzed. The indices that were estimated using HF SNPs showed a slight upward trend between 1970 and 2009, followed by a slight reduction in diversity during the last period (2010–2020). However, the analysis of LF SNPs showed a different pattern, increasing from 1915 to1979, followed by a three-fold downward trend in diversity to the present (Additional file 3: Figure S1c,d). Despite this, the highest number of LF PAs was observed between 2000 and 2009, with 590 PA (52.6%) followed by 396 PA in 1970–1979 (35.3%) (Table 2). The highest average number of PAs by accession was found in the period 1970–1979 (28.3 PA). The estimated Nei genetic distance among breeding periods showed the highest differentiation between the 1960–1969 and 2010–2020 periods (Additional file 5: Table S4b).

Only 15 genotypes of the collection captured most of the rare allelic variants, in particular the cultivar Polesine (ITT, 1970–1979) and the Chilean breeding line Quc 3506–2009 (2000–2009) that carried more than 200 PA (Additional file 4: Table S3c).

### Linkage disequilibrium patterns

**Fig. 1:**
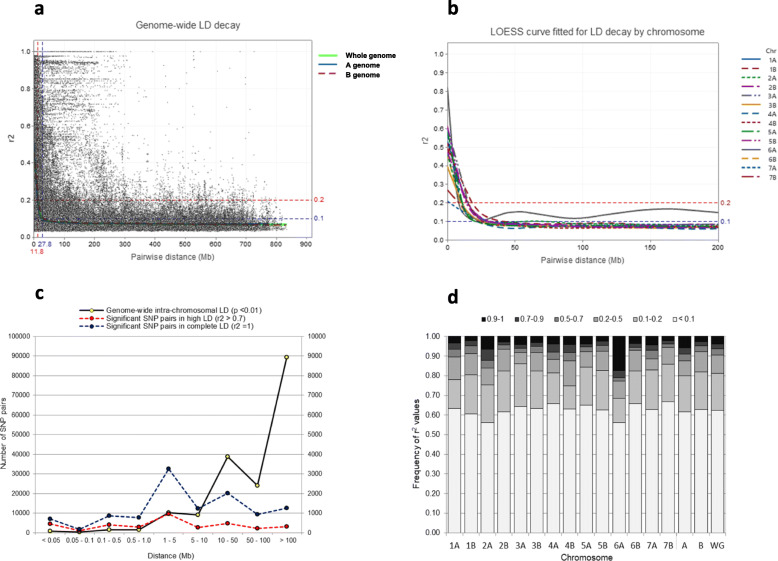
Genome-wide linkage disequilibrium (LD) distribution and LD decay. **a** Scatter plot of LD values of intra-chromosomal pairwise loci against physical distance (Mb). LD decay was fitted with the locally weighted polynomial regression-based (LOESS) curve by genome and for genome-wide LD. **b** LOESS curves fitted by chromosome (only distance to 200 Mb is shown); **c** Number of SNP pairs in LD distributed along physical distance intervals; d) LD (*r*^*2*^) values frequency by chromosome, genome and whole genome

Analysis of genome-wide LD in the whole collection showed that 13.37% of the total marker pairs had a significant LD (*p* < 0.01), with a mean *r*^*2*^ value of 0.0895. Only 4.74 and 0.95% of the significant marker pairs showed *r*^*2*^ values above 0.2 and 0.7, respectively, indicating a low level of LD in the genome. Differences in the significant intra-chromosomal LD were observed between the A and B genomes resulting in higher values in the A genome. Analysis of variance detected significant differences (*p* < 0.001) in LD between chromosomes, with the 6A chromosome having the highest mean *r*^*2*^ value (*r*^*2*^ = 0.290), followed by 2A, 4B, 1A, 4A and 7A. Moreover, the 6A had the lower proportion of significant marker pairs in LD (15.1%), whereas the highest value was observed in the 1B chromosome (27%) (Table 1). The frequency of *r*^*2*^ values in each chromosome is shown in Fig. 1d.

The distribution and extent of LD were displayed as decay plots and a second-degree locally-weighted polynomial regression (LOESS) curve was fitted by chromosome, each genome and for the whole genome (Fig. 1a, b). The critical threshold *r*^*2*^ value, corresponding to the 95th percentile of the distribution of the square root transformed inter-chromosomal LD, was *r*^*2*^ = 0.196, very close to the 0.2 suggested by [44]. The intra-chromosomal LD decay, below an *r*^*2*^ critical threshold lower than 0.2, showed a mean value of 11.8 Mb in the whole genome below which the LD is probably caused by a real physical linkage. The LD decay varied from 5.6 (7A) to 19.1 (1B) Mb in the chromosomes (Table 1, Fig. 1a, b). Beyond the inter-marker distance indicated as whole genome LD decay, 88.2% of the *r*^*2*^ values were below 0.2 and only 4.4% were values higher than 0.5. Alternatively, the LD decay was calculated as the variation of the mean *r*^*2*^ value across distance in each chromosome [45] (Additional file 6: Figure S2a).

LD decay was also calculated considering the Argentinian germplasm only, obtaining values of 60.6 Mb for the A genome, 34.7 Mb for the B genome and a whole genome value of 30.4 Mb which is 2.5 fold higher than the one obtained when the whole collection was considered (Additional file 6: Figure S2b, c, d). The mean *r*^*2*^ values for the Argentinian germplasm and by chromosome are also shown in Table 1.

On the other hand, the number of marker pairs, in high (*r*^*2*^ > 0.7) or complete LD (*r*^*2*^ = 1), was assessed for each chromosome and its distribution considering the inter-marker distance was evaluated. As a result, the percentage of marker pairs in complete intra-chromosomic LD (*r*^*2*^ = 1) in the whole genome was very low (1.97%). The 2A, 6A, 1B, 2B, 7A chromosomes showed the highest number of marker pairs in complete LD, whereas 1B, 2A, 6A, 7A and 2B exhibited the highest number in high LD (*r*^*2*^ > 0.7). This analysis was repeated taking into account only the Argentinian germplasm being the number of marker pairs in high LD (*r*^*2*^ > 0.7) 11.7% higher and the complete LD (*r*^*2*^ = 1) 88.9% higher than in the whole collection, in particular for the 6A, 2A, 7A and 1B chromosomes (Additional file 6: Figure S2e, f).

Considering the whole genome, the number of pairwise SNPs showing high (*r*^*2*^ > 0.7) or complete LD (*r*^*2*^ = 1) values was maximum in an inter-marker distance range of 1 to 5 Mb (Additional file 6: Figure S2g, h). However, different behavior was observed in three chromosomes (2A, 7A and 6A) showing an increasing number as the distance between pairs of SNPs increased, suggesting a higher extension of high LD in these chromosomes. The 1B chromosome exhibited extended high LD between 1 and 50 Mb, also shown in Additional file 6: Figure S2d. LD heat maps by chromosome and for whole genome revealed larger LD blocks on chromosomes 6A, 4B, 2A, 7A, 4A, 1B, 1A and 3B (Additional file 7: Figure S3a, b).

In addition, the inter-marker distance estimated considering the SNP pairs in complete LD was higher in the Argentinian germplasm compared with the whole collection values (Table 3).

**Table 3:** Mean inter-marker distance for SNP pairs in complete LD (*r*^*2*^ = 1)

Chr. / Genome	Whole collection	Argentinian accessions
1A	7.38	6.57
1B	14.93	10.52
2A	69.79	81.28
2B	5.63	5.48
3A	8.07	14.34
3B	3.66	17.84
4A	6.69	8.68
4B	3.13	3.00
5A	5.56	10.79
5B	0.91	2.04
6A	29.50	57.48
6B	9.07	19.84
7A	41.40	51.93
7B	23.95	30.72
A genome	35.13	53.96
B genome	7.46	13.11
Whole genome	25.12	37.79

*Chr.* chromosome

**Fig. 2:**
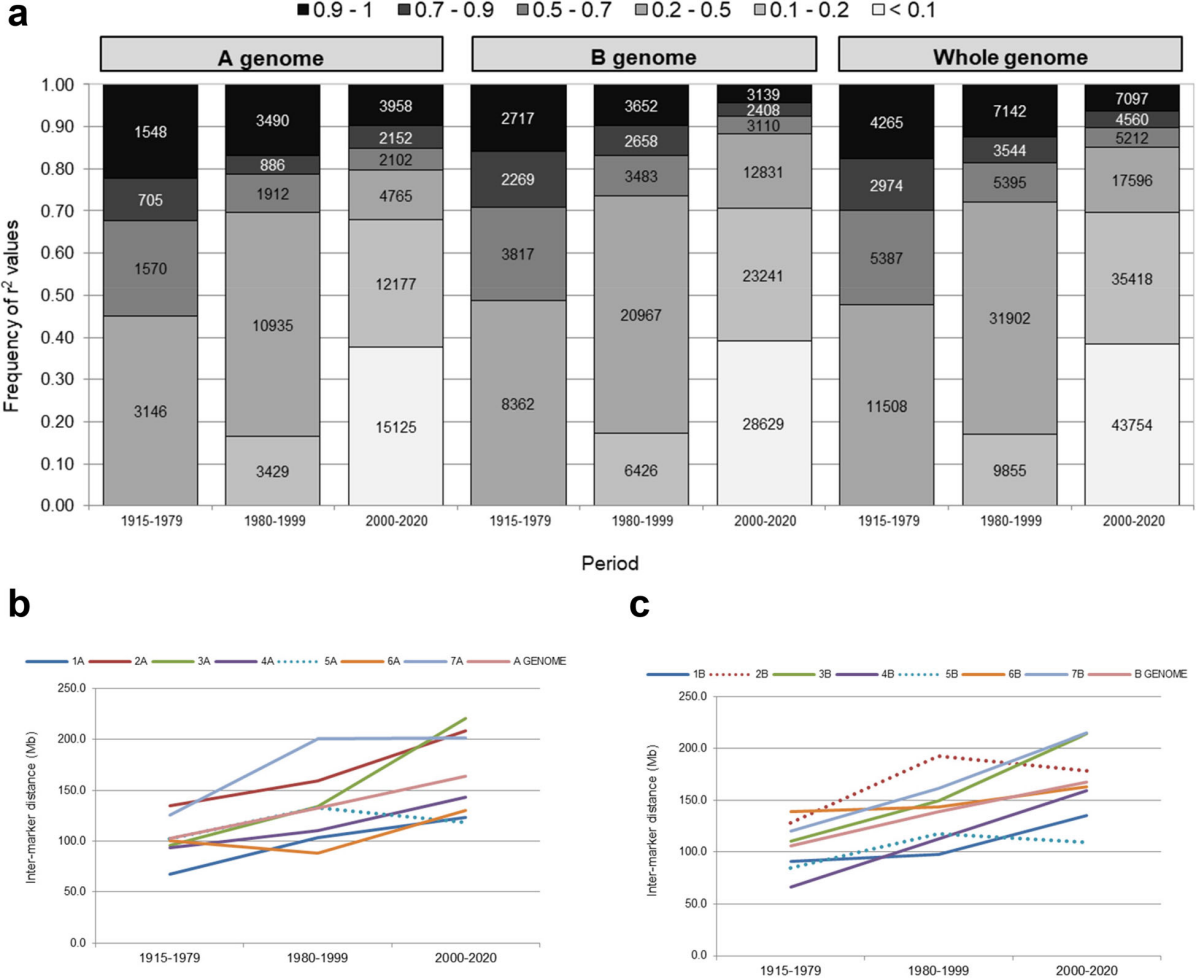
Comparison of LD distribution in three breeding periods (**a**). Changes of average inter-marker distance in significant LD (*p* < 0.01) over time assessed by chromosome (**b**) and genome (**c**)

An overall increase over time in significant LD, and also in the extension of LD measured as an average of inter-marker distance (Mb) (Fig. 2), was observed as an effect of breeding, considering three main periods (1915–1979, 1980–1999 and 2000–2020). In this sense, the number of pairwise SNPs in high LD (*r*^*2*^ > 0.7) increased over time, but the proportion of these markers decreased as a consequence of an overall increase in the background LD. Different LD patterns in the A and B genomes and in the chromosomes were observed over time (Additional file 8: Figure S4). In general, the SNP pairs on the B genome in high LD decreased between the second and third periods. The 6A chromosome was the only one showing an increase in the number and a proportion of pairwise in complete LD = 1 simultaneously over time.

### Population stratification and diversity

The population structure was studied in our collection using a subset of 675 markers selected from the complete dataset. These markers were almost evenly distributed throughout the whole genome (Table 1).

**Fig. 3:**
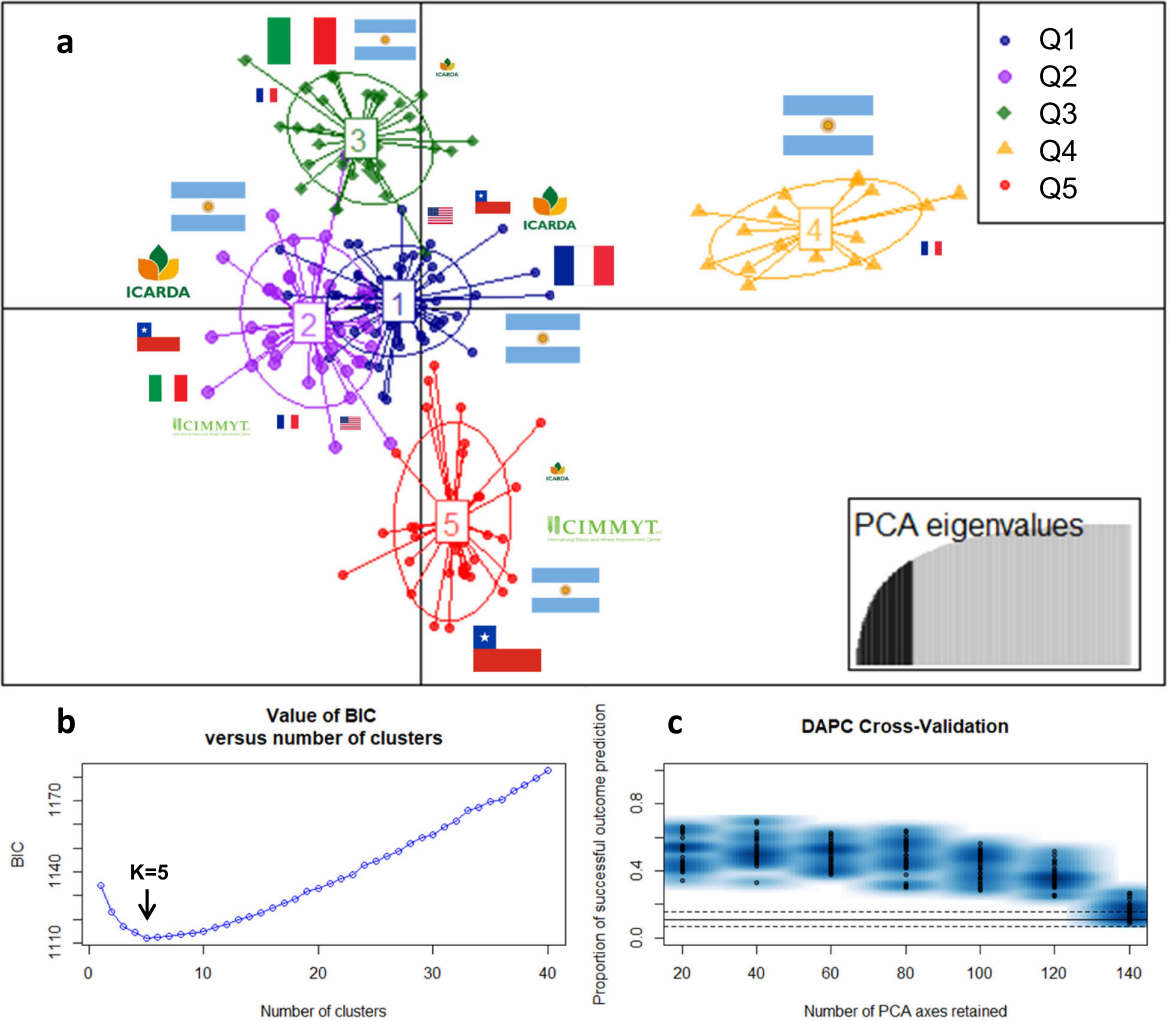
Population structure according to the discriminant analysis of principal components (DAPC) using 675 SNPs. The first two components are displayed graphically (each sub-population is differentiated by color) (**a**). Cluster selection was based on the BIC value (**b**). Number of PC retained using the cross-validation test (**c**)

Five sub-populations were inferred by the Discriminant Analysis of Principal Components (DAPC) based on BIC criterion (Fig. 3). For this analysis, 40 PCs were retained using the cross-validation method. The modern Argentinian germplasm was mainly distributed in four sub-populations, Q1 (28), Q2 (16), Q4 (16) and Q5 (9), indicating the high diversity present in this germplasm. The only modern Argentinian cultivar included in Q3 was BonINTA Cumenay. Three traditional Argentinian accessions were included in Q1, one in Q2, nine in Q3 and only one in Q4.

The sub-population Q1 mostly included modern Argentinian accessions (28), most of the French germplasm (19 out of 22) and intermediate contributions of WANA (6), Chile (4), traditional Argentinian (3) and modern Italian accessions (3). Two out of the three Argentinian breeding programs included in this study (INTA and ACA) made a major contribution to this group and 72% of the germplasm included in Q1 corresponded to the last two breeding decades. Among these contributions the Argentinian cultivar BonINTA Carilo was widely present in the pedigree of the breeding lines of this sub-population. The U.S. cultivar Kofa was also included in this group, as well as several breeding lines from the Argentinian program of ACA which frequently used Kofa as a parental line for end-use quality traits.

The sub-population Q2 included 16 Argentinian accessions, followed by nine from WANA, five from Chile, three from CIMMYT and three modern Italian genotypes. This sub-population showed greater influence in the pedigrees of accessions from the CIMMYT/ICARDA breeding programs. The Q2 cluster included four Om Rabi accessions and its parental line Haurani, all from the WANA region. The founder genotypes Altar 84 (Gallareta) and Yavaros-79 (Chagual INIA), two genotypes widely used by CIMMYT in different breeding programs, were also included. The cultivar Buck Topacio (PROB611/Altar 84) belongs to this sub-population, cultivated in Argentina for 20 years, together with derivative breeding lines from INTA and BUCK Semillas.

The sub-population Q3 was mainly composed of Italian germplasm (24 of 36), i.e. 15 out of 17 traditional, and nine modern, Italian accessions. This sub-population also includes nine out of the 14 traditional Argentinian accessions and it is mostly composed of old genotypes (58%), released between 1915 and 1979, with great influence of Cappelli and Taganrog, two founder genotypes. The only modern Argentinian genotype included in Q3 (BonINTA Cumenay) is mainly a derivative of the last two mentioned genotypes. In addition, here were included all the accessions from the Gerardo group (GIORGIO//CAPELLI/YUMA).

The fourth subpopulation (Q4) was the smallest group (18) inferred by DAPC, mostly corresponding to 16 modern and one traditional Argentinian (Buck Candisur, from 1982) and one French accessions (Arcodur). This cluster mainly included germplasm from the BUCK breeding program, or breeding lines from INTA, but carrying a genetic derivative from BUCK Semillas. Eighty three percent (83%) of the germplasm included in Q4 was developed in the last 20 years. In addition, the pedigree analysis showed a wide use of the cultivar Buck Ambar as part of these crosses.

Pedigree analysis showed that the sub-population Q5 included accessions with the greatest influence of CIMMYT germplasm, mainly bred or released during the 2000–2020 period. This group includes most of the Chilean breeding lines (17) and two recently released cultivars, Lleuque INIA (2011) and Queule INIA (2014). This group was also composed of 10 Argentinian accessions and germplasm from CIMMYT nurseries (6).

**Fig. 4:**
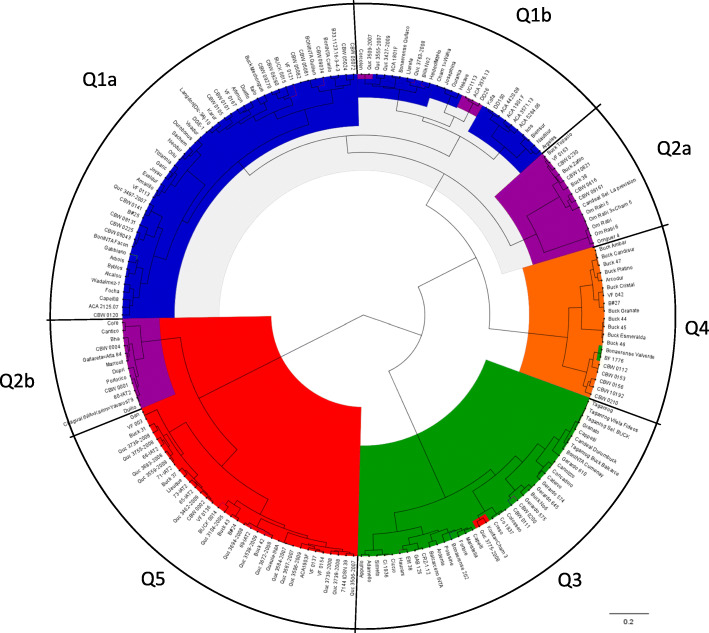
Phylogenetic relationships based on genetic distance in 197 durum wheat accessions displayed graphically using a Ward dendrogram. The sub-populations found by DAPC are indicated with colors and named on the external circle

Population structure was also studied using the Bayesian model-based method implemented in STRUCTURE software. In contrast to DAPC, this analysis obtained a maximum ΔK at K = 2, indicating less ability to discriminate the sub-populations clearly. At K = 2 the sub-population Q1_K2 with 85 accessions was mainly composed of germplasm with the greatest CIMMYT contribution, including 30 modern Argentinian genotypes, all the Chilean accessions (26), 10 CIMMYT cultivars or breeding lines, and half (9) of the WANA region accessions. On the other hand, the sub-population Q2_K2, consisting of 112 genotypes, included 41 modern and 12 traditional Argentinian accessions and most of the Italian, French, American and half of the WANA region accessions (detailed in Additional file 9: Table S5a). Population stratification was tested by the genetic distance-based method, followed by a Ward hierarchical clustering implemented in the DARWIN v6.0 software (Fig. 4). This analysis was able to establish the genetic relationships between accessions and also to detect the main sub-populations previously identified using DAPC. Based on pedigree information, sister lines (such as Buck 44, Buck 45 and Buck 46) and breeding lines with their parental lines were clustered together, as for example BonINTA Carilo and their derivatives.

The number of sub-populations defined a priori by DAPC (K = 5) and the two additional methods were compared. The Ward clustering method divided Q2 (DAPC) into two main blocks, Q2a more closely related to Q1 and Q2b clustered proximal to Q5. Q2b represented genotypes with major influence of CIMMYT, including the founder genotypes Altar-84 and Yavaros-79. When the convergence of these three methods was analyzed a clear pattern in the assignment of accessions to Q3, Q4 and Q5 was observed, showing several differences between Q2 from DAPC and Ward clustering and between Q1 from DAPC and STRUCTURE (detailed in Additional file 9: Table S5b). The results of a comparison between the Ward clustering method and STRUCTURE are graphically displayed in Additional file 10: Figure S5.

The Analysis of Molecular Variance (AMOVA) considering the five DAPC genetic sub-populations revealed that 22% of the variation (*p* < 0.001) was between sub-populations and 78% was intrapopulation. In addition, the pairwise *Fst* index showed that the Q5 (CIMMYT derivatives) and Q3 (mostly Italian germplasm or derivatives) (*Fst* = 0.337) sub-populations showed the most difference. Q4 with Q3 and Q4 with Q5 also exhibited high pairwise *Fst* and *He* values, but lower than the former mentioned pair of sub-populations. (see Additional file 5: Table S4c).

The Q1 sub-population from DAPC showed the highest Nei’s gene diversity index (*He*), followed by Q2 and Q3. Using HF SNPs only two private alleles were detected, one in Q1 and another in Q4. Considering the LF SNPs, also called rare alleles, the highest diversity (as *He* and *I* index) and number of private alleles were observed in Q3, followed by Q1 and Q5.

## Discussion

### Distribution and physical location of polymorphic SNPs

In this study a total of 7431 polymorphic SNPs were assessed in the 14 durum wheat chromosomes using a collection of 197 accessions, facilitated by the availability of high quality genome assemblies that allowed the co-localization of markers [37–39]. SNP loci in annotated genes (Svevo genome assembly) were verified, considering both the LF SNPs and the HF SNPs. In both cases, SNPs were found in the annotated genes, representing 20.8% of the total polymorphic SNPs (23.1% LF and 19.6% HF SNPs). In most cases only one SNP was located on a gene (80% of SNPs alignments). However, in some genes several polymorphic SNPs (up to nine) could be found. According to [46], allelic rare variants could contribute to complex disease resistance and discarding them might reduce the chance to find associations with disease resistance traits. In our study 16 SNPs with rare alleles and 43 HF SNPs were found in annotated disease resistance genes (NBS-LRR domains and other). An increase in the frequency of rare alleles in breeding programs introduced from landraces or related wild species is a commonly adopted method for gaining variability [47, 48]. The parallel identification of allelic variations for known functional genes contributes to the efficient use of genetic resources for widening genetic diversity in elite wheat lines.

Furthermore, the results showed other annotated genes carrying polymorphic SNPs aligned as the *Gli-B1* locus encoding for gamma-gliadin in 1BS chromosome ([LF], AX-94659353), the *HYD-B1* locus encoding the carotenoid β-hydroxylase 1 gene ([HF], AX-94475906) in the 2B chromosome, a soluble starch synthase gene (*ss3*) located in 1A (two LF SNPs, AX-95209651 and AX-94805209) and a lipoxygenase gene putatively encoding the *Lpx-A2* locus in chromosome 5A ([HF], AX-94964352), all important genes associated with relevant quality traits in durum wheat breeding programs [49–52].

### Genetic diversity

The genetic diversity using HF SNPs assessed in our collection was moderate (*I* = 0.503, *He* = 0.333), but acceptable considering the bi-allelic nature of the SNPs. Lower indices were reported for 259 genotypes (*I* = 0.38, *He* = 0.24) included in a durum wheat collection (old and modern Italian cultivars and landraces) by [17]. Also, [53] reported lower values (*He* = 0.228) using 150 durum wheat landraces and cultivars from 1901 to 2009. Genetic diversity based on the number of low frequency SNPs was also assessed. The number of rare related alleles has been directly associated with the allelic richness in subdivided populations [54] and it is also considered as an indicator of gene flow between sub-populations [55]. Allelic richness is an alternative criterion for measuring genetic diversity and is considered a key parameter for germplasm conservation programs [56]. Loss of rare alleles could be associated with genetic erosion and decreased long-term adaptation [57]. The use of a low number of parental lines or the recurrent use of cultivars in breeding produces a narrowing of the genetic base and could be responsible for a founder effect or loss of allele richness in the segregating germplasm. This was observed in the Q4 sub-population which exhibited the lowest percentage of polymorphism in LF SNPs.

Based on geographical origin, the modern Argentinian genotypes exhibited the highest level of genetic diversity (*H*e or *I* index) followed by French and traditional Italian genotypes when HF SNPs were used. The traditional Italian genotypes exhibited the highest diversity (*He*) estimated using the LF SNP and also the highest number of private alleles. The modern Argentinian accessions showed a slightly higher *He* value (for HF SNPs) and a higher number of private alleles (for LF SNPs) than the modern Italian genotypes. The decreased diversity observed using both HF and LF SNPs from traditional and modern Italian accessions agrees well with the results of [19]. Similar values of Nei and Shannon indices in the old and modern Italian cultivars were reported by [17].

Genetic diversity over a timeline (using HF SNPs) showed a slight increased (5.4%) between the 1970–1979 and 2000–2009 periods but decreased during the latest one (2010–2020) returning to a similar *He* value as in the 1970–1979 period. On the contrary, [13] only considering Canadian germplasm, reported a decline in diversity from 1950 to 2010. In the same way, genetic diversity decreased during the development of the ICARDA breeding program from 1974 to 2007 [58]. Interestingly, even though a reduction in Nei’s diversity index, using LF SNPs, was observed between the 1970–1979 and 2000–2009 periods, the number of LF and private alleles was higher in 2000–2009 than in the other periods. According to [59] the number of alleles per locus detected in a finite population depends on the effective population size. This concept could explain the high number of LF SNPs captured in the 2000–2009 period which exhibit the highest number of genotypes. It is promising to observe that allelic richness is preserved within the more recent germplasm. This period included most of the modern Argentinian, Chilean and French genotypes. A decrease in genetic diversity estimated using LF SNPs was also observed by [58], suggesting that effective strategies to incorporate and increase the amount of these variants should be addressed. Recent studies in wheat associated LF SNPS (rare alleles) with larger grains [60], or improvements in grain size and yield in rice [61]. These alleles could also be used to trace the degree of genetic contribution in different sub-populations [62] and could have long-term implications in the adaptive response towards environmental changes [57]. Otherwise, short-term response to selection is highly dependent on additive genetics estimated as expected heterozygosity (*He*) [63]. Accordingly, this study provides evidence to define genetic diversity strategies in breeding programs aiming to maximize both heterozygosity and allelic richness in order to obtain a rapid short-term response to selection and producing more resilient wheats.

### LD patterns

The extent and distribution of linkage disequilibrium in the genome define regions that are inherited together [64]. Our analysis detected 13.4% of the total marker pairs with significant LD (*p* < 0.01), a considerably lower percentage in comparison with the 42% (*p* < 0.001) obtained by [15] and the intra-chromosomal LD values reported by [48, 65, 66]. In our collection, high LD (*r*^*2*^ > 0.7) was only represented in 0.94% of significant pairwise comparisons, being four-fold lower than the value reported by [67]. Considering a threshold of the 95th percentile of the root transformed *r*^*2*^ value distribution (*r*^*2*^ = 0.196), the LD decay (11.8 Mb) detected in our collection was acceptable for modern cultivars and it was similar to the values obtained in other durum wheat panels (9.6 Mb in [15] and 9.96 Mb in [17]), but lower than the distance (51.3 Mb) reported by [68] and by [41] (21 cM) or by [42] (14 cM). However, for breeding purposes the estimation of LD decay in local germplasm could be useful due to the differences between regional germplasm and large populations, as was observed between the Argentinian accessions and the whole collection where we could observe a 2.5 fold higher LD decay values. An increase in the mean inter-marker distance was also observed in the SNP pairs in complete LD.

According to [69], the trend in LD decay could be described using different estimators or functions. In the present study, the number of SNP pairs in high (*r*^*2*^ > 0.7) or complete LD (*r*^*2*^ = 1) over distance was described, finding the highest number of significant pairs in LD between 1 and 5 Mb. A different pattern was observed when overall LD was considered. Some authors reported the presence of local epistasis in winter wheat [70], but the LD pattern could vary substantially with the population [40]. Some authors indicate that high LD between closely linked loci can be created by genetic drift, bottlenecks or selection [70]. However, some chromosomes, such as 2A and 7A, showed an extended and rising number of SNPs in high or complete LD as a function of distance, probably due a differential selection pressure exerted on these chromosomes to maintain agronomically advantageous or epistatic loci during the breeding process [40, 71]. In the overall LD, the number of SNP pairs in significant LD became higher as the pairwise distance increased (presented in Fig. 1c). In addition, the LD patterns between neighboring loci were also analyzed by plotting heat maps and several LD blocks were observed on the 6A, 1B, 4B, 2A, 7A, 1A and 3B chromosomes. Long-range LD blocks on 1B and 6A chromosomes were also reported by [72] and signatures of selection based on LD on 1B and 7A were observed by [73]. Some of these regions could correspond to the putative position of major known genes in wheat, such as dwarfism genes *Rht-1* (4B and 4A, [74]), the photoperiod sensitivity gene *Ppd-A1* (2A, [75]), the glutenin loci *Glu-2*/*Glu-3* and *Glu-1* (1B and 1A, [49, 76], the Gliadins *Gli-2* and *TaGW2* loci (6A, [77, 78]) or *TaSus1* (7A) (https://wheat.pw.usda.gov/GG3/node/759 [39, 79];).

LD patterns assessed over three main breeding periods (1915–1979, 1980–1999 and 2000–2020) demonstrated an increased number of overall pairwise LD over time. Even though, a reduction in the mean *r*^*2*^ value was observed over the three periods due to a dilution effect caused by a higher proportion of background LD (*r*^*2*^ < 0.5). The occurrence of high background LD was supported by an increased average inter-marker distance in most of the chromosomes over time. Chromosomal LD pattern over time suggested that breeding and selection have impacted differently on the A and B genomes. In our collection, the SNPs with high or complete LD decreased from 1980 to 1999 to 2000–2020 in the B genome, but consistently increased over time in the A genome. The highest effect of artificial selection over time was observed for the 6A chromosome. Previous reports also indicated differences in LD patterns in the A, B and D genomes in bread wheat [42, 48, 80, 81].

### Population structure

Population stratification can occur as a consequence of artificial selection in breeding, parental bottlenecks, geographical origin of germplasm and genetic drift [82–85]. Population structure using different methods was applied, as suggested by [86]. We used the Bayesian model-based method implemented in STRUCTURE, a nonparametric method, as DAPC and a distance-based clustering method (Ward).

This study evaluated 111 South-American durum wheat accessions (mainly from Argentina and Chile), including also additional world-wide genotypes. Our results indicated the existence of five sub-populations with moderate to high differentiation (*Fst* ranging from 0.139 to 0.337), slightly higher than the one reported by [13], but lower than that of [17] which included a large collection of landraces. The AMOVA assessed considering these sub-populations explained about 22% of the variance between groups, a lower value than reported by [12]. The DAPC and Ward clustering results showed that the modern Argentinian germplasm combines contributions from different genetic sources, such as Mediterranean genotypes (Q1 and Q2), or accessions from CIMMYT (Q5 and partially in Q2). An interesting result was the evidence that a part of the modern Argentinian accessions (Q4) was clearly differentiated from the remaining sub-populations, mostly germplasm from BUCK Semillas company. This finding confirms previous results obtained by our group based on 26 SNPs (KASP™) that gave indications of a possible genetic differentiation [14]. On the other hand, the STRUCTURE results were able to differentiate clearly only the K = 2 as the main stratification level, based on ΔK parameter. However, considering the clustering of entries at the K = 5 level this result gave up to 77% of coincidence with DAPC (100% in Q3 and Q4 as it is shown in Additional file 10). The use of different methodologies as suggested [86] contributed to better understanding of the genetic relationships between the accessions and lets to infer that the cluster Q4 have greater similarity with the Mediterranean germplasm.

In addition, there was evidence of a founder effect of Buck Ambar in this modern Argentinian germplasm (Q4). Whereas that another two Argentinian cultivars, BonINTA Carilo (Q1) and Buck Topacio (Q2), were widely used in crosses and their derivative lines were mostly clustered together with the parental lines. In comparison, the ACA Coop Ltda. breeding program extensively used the desert durum cultivar Kofa, which was clustered with its derivative breeding lines in Q1. Considering all the methodologies used in this study, population structure analysis also divided the traditional or old germplasm, mostly included in Q3 (≈75% bred before 1989), from the modern ones. Most of the landraces (Taganrog, Etit 38) and the old cultivar Cappelli were also included in Q3, except for Haurani which was only clustered in Q3 when using the Ward clustering method. A gradient in the contribution of CIMMYT germplasm from Q5, passing through Q2 and finally to Q1 was shown from the three sub-populations with a major influence of the CGIAR durum wheat breeding programs. The WANA region germplasm, with greater influence or derived from ICARDA, was mostly represented by Q2 and Q1. In general, population structure analysis corroborates the previous pedigree information and the a priori relationships between parental lines with derivative lines and between sister lines.

## Conclusions

The development of national breeding programs of durum wheat in Argentina began with the introduction of European germplasm, local breeding and subsequent incorporation of CIMMYT germplasm during the green revolution era. Three main national breeding programs have been permanently maintained over the last 50 years. Nowadays, some international companies have recently established breeding programs or released introduced cultivars. The present study demonstrated that the breeding germplasm developed in Argentina is the result of an admixture from different genetic sources. An important highlight is that selection patterns and diversity structure were identified in the germplasm subgroups resulting from decades of locally adapted breeding. Rare alleles can be used as sources of variability and may provide favorable alternatives for facing future challenges.

From a breeding approach, the selection of a strategy for increasing allelic richness based on adaptive potential in the segregating germplasm is essential under a climate change scenario. Recent international effort has resulted in dynamic platforms or initiatives for sharing genetic resources that guarantee free germplasm exchange and permit a continual widening of the genetic base in breeding. On the other hand, the study of LD indicated that selection pressure during breeding has impacted differently on chromosomes resulting in differences in the extension and level of LD and haplotypes. This should be considered at the time of applying marker assisted selection.

## Methods and materials

### Plant material

A durum wheat (*Triticum turgidum* L. ssp*. durum* Desf. Husn) collection composed of 197 worldwide accessions (landraces, cultivars and breeding lines), including 168 genotypes previously described by [14], was used for this study. This collection is mostly representative of the Argentinian breeding programs (85), but also includes accessions from Italy (33), Chile (26), France (22), WANA (17), CIMMYT (10) and the USA (4) (Additional file 1: Table S1a). Both the Italian and Argentinian accessions were classified as ˋtraditional´ or ˋmodern´ (before and after 1985) based on previous results which detected an association of origins according the breeding period [14].

### SNP genotyping and data filtering

DNA from each accession was extracted from fresh leaves of 10-day-old seedlings using a modified CTAB method, as described in [87]. The durum wheat collection was genotyped using the 35 K Axiom Wheat Breeder’s Genotyping Array from Affymetrix [33] at TraitGenetics (Gatersleben, Germany) and CCT CONICET La Plata (Argentina). The SNP matrix was filtered, discarding the monomorphic markers, SNPs with > 10% of missing data and SNP with > 10% of heterozygosity. Markers with minor allele frequency (MAF) < 0.05 were analyzed separately to study the diversity due to LF SNPS. The SNPs classified as polymorphic high resolution (Poly High-resolution) and off-target variant (OTV) having good cluster resolution were considered, following the recommendations for polyploid species of Axiom® Genotyping Solution Data Analysis Guide (http://www.affymetrix.com/). The OTV SNPs were analyzed with the OTV-caller function before use.

### Distribution and physical positions of polymorphic SNPs in the durum wheat genome

The physical positions of the SNPs were obtained by BLASTN [88] of each SNP sequence on the durum wheat reference genome assembly (Svevo CV) (https://wheat.pw.usda.gov/GG3/node/759), with a threshold of 95% for identity and coverage. Additional information of the SNP positions were obtained from nulli-tetrasomic lines (https://www.cerealsdb.uk.net), BLASTN results on bread wheat (http://plants.ensembl.org/Triticum_aestivum/Info/Index), wild Emmer wheat genomes (https://wewseq.wixsite.com/consortium) and genetic positions on published linkage maps [33, 89, 90], especially if the SNPs showed multiple hits (homeologous or interchromosomic duplication). The SNPs positioned on the Svevo genome assembly were used to identify polymorphisms in annotated genes (http://plants.ensembl.org/Triticum_turgidum/Info/Index).

### Genetic diversity

Basic genetic statistics were calculated using the GenAlex v6.5 software [91, 92] to describe genetic diversity including the percentage of polymorphic loci (%PL), observed heterozygosity (*Ho*), Nei’s gene diversity (PIC=*He* = expected heterozygosity) [93, 94] and Shannon’s information index (*I*) [95]. The fixation index (*Fst* = (Ht-Hs)/Ht) or genetic differentiation in populations index [96] was calculated between sub-populations detected by Discriminant Analysis of Principal Components (DAPC).

Polymorphic SNP markers that passed quality controls but showed an MAF < 0.05 (LF), commonly called “rare alleles”, were used to identify subgroups in the collection that could be considered as a reservoir of genetic diversity. These rare alleles are referred to as private alleles (PA) when only found in a single subgroup of a broader collection. Predefined groups according the country/region of origin (Argentinian traditional [ART], Argentinian modern [ARM], Chile [CHI], CIMMYT (México) [CIM], France [FRA], Italian traditional [ITT], Italian modern [ITM], United States [USA], West Asia/North Africa region [WANA]), the sub-populations from structure results and the ranges of periods of bred/released (1915–1959, 1960–1969, 1970–1979, 1980–1989, 1990–1999, 2000–2009, 2010–2020) were considered as subgroups for testing genetic differences.

### LD estimation and LD decay

Linkage disequilibrium (LD) was calculated in the TASSEL 5.0 software [97] considering only SNPs with an MAF > 0.05 to avoid a bias effect of the LF SNPs on LD [98]. LD was measured as the allele frequency correlation (*r*^*2*^) for all pairwise SNP comparison in each chromosome and subsequently the chromosome and genome specific mean values were estimated. Inter-chromosomic LD (unlinked loci) was estimated over the whole genome. The LD decay was determined by plotting the *r*^*2*^ values against the genetic distance of loci pairs (Mb) for each chromosome and a trend line describing the LD decay was calculated by locally-weighted polynomial regression (LOESS) in R (http://www.r-project.org). The 95th percentile of the distribution of square root transformed inter-chromosomal LD values (*r*^2^) [99] was estimated as the critical threshold below which the LD could be considered as being caused by physical linkage. The intersection point between the LD curve and the *r*^*2*^ threshold determined the LD decay value for each chromosome.

### Population structure

To assess the population structure, the SNPs were selected by considering inter-marker distances greater than 1 Mb and MAF > 0.3 to select informative and well distributed markers according to the recommendations of [86]. The population structure was explored by the Discriminant Analysis of Principal Components (DAPC) method implemented in the R package “adegenet” v2.0.1 [100] in R studio V 1.3.1056 (R Development Core Team, 2011). The number of PC retained was selected by the cross-validation method using the *xvalDapc* function. The most probable K was declared, based on the lowest Bayesian Information Criterion (BIC) value following the [10] criteria. In addition, population structure was analyzed using the STRUCTURE v2.3.4 software (http://pritch.bsd.uchicago.edu/structure.html), selecting the admixture as the ancestry model and the correlated allele frequencies option [101]. Parameters were set at 100,000 burning periods and 100,000 Markov Chain Monte Carlo (MCMC) replicates using 5 independent runs for each K (1 to 10). No prior information was provided. The Evanno test [102] was used to identify the true number of sub-populations (K) implemented in the STRUCTURE HARVESTER website [103]. STRUCTURE results were plotted using the Pophelper 2.3.0 R library [104]. Furthermore, the Ward clustering based-distance method was used to assess the genetic relationships between the accessions, based on a dissimilarity index calculated from the simple matching coefficient in DARwin v6.0 software [105]. The Ward dendrogram was drawn in the FigTree v1.4.3 software (http://tree.bio.ed.ac.uk/software/figtree/).

## Supplementary Information

**Additional file 1.**

**Additional file 2.**

**Additional file 3.**

**Additional file 4.**

**Additional file 5.**

**Additional file 6.**

**Additional file 7.**

**Additional file 8.**

**Additional file 9.**

**Additional file 10.**

## Data Availability

Plant material and raw data are available upon request to the first author. Annotated genes are public available in http://plants.ensembl.org/Triticum_turgidum/Info/Index. Phylogenetic tree, dissimilarity matrix and dataset to obtain them are available in http://purl.org/phylo/treebase/phylows/study/TB2:S27474.

## Abbreviations

CIMMYT: Centro Internacional de Mejoramiento de Maíz Y Trigo
CARDA: International Center for Agricultural Research In the Dry Areas
INIA: Instituto de Investigaciones Agropecuarias
CCT: Centro Científico Tecnológico
INTA: Instituto Nacional de Tecnología Agropecuaria
ACA: Asociación de Cooperativas Argentinas
LD: Linkage Disequilibrium
SNPs: Single Nucleotide Polymorphisms
KASP: Kompetitive Allele Specific PCR
MAF: Minor Allele Frequency
HF: High Frequency
LF: Low Frequency
PA: Private Alleles
LOESS: Locally-weighted polynomial regression
DAPC: Discriminant Analysis of Principal Components
AMOVA: Analysis of Molecular Variance
CGIAR: Consultative Group on International Agricultural Research
CTAB: Cetyl Trimethyl Ammonium Bromide
BIC: Bayesian Information Criterion
PCoA: Principal Coordinate Analysis
