# Supplementary material for: Linkage disequilibrium patterns, population structure and diversity analysis in a worldwide durum wheat collection including Argentinian genotypes
Source: BMC Genomics. 2021 Apr 5;22:233. doi: 10.1186/s12864-021-07519-z (PMC8022437; doi:10.1186/s12864-021-07519-z)
Supplement: Supplementary file 7 — Additional file 7. [file 12864_2021_7519_MOESM7_ESM.pptx]

## Slide 1
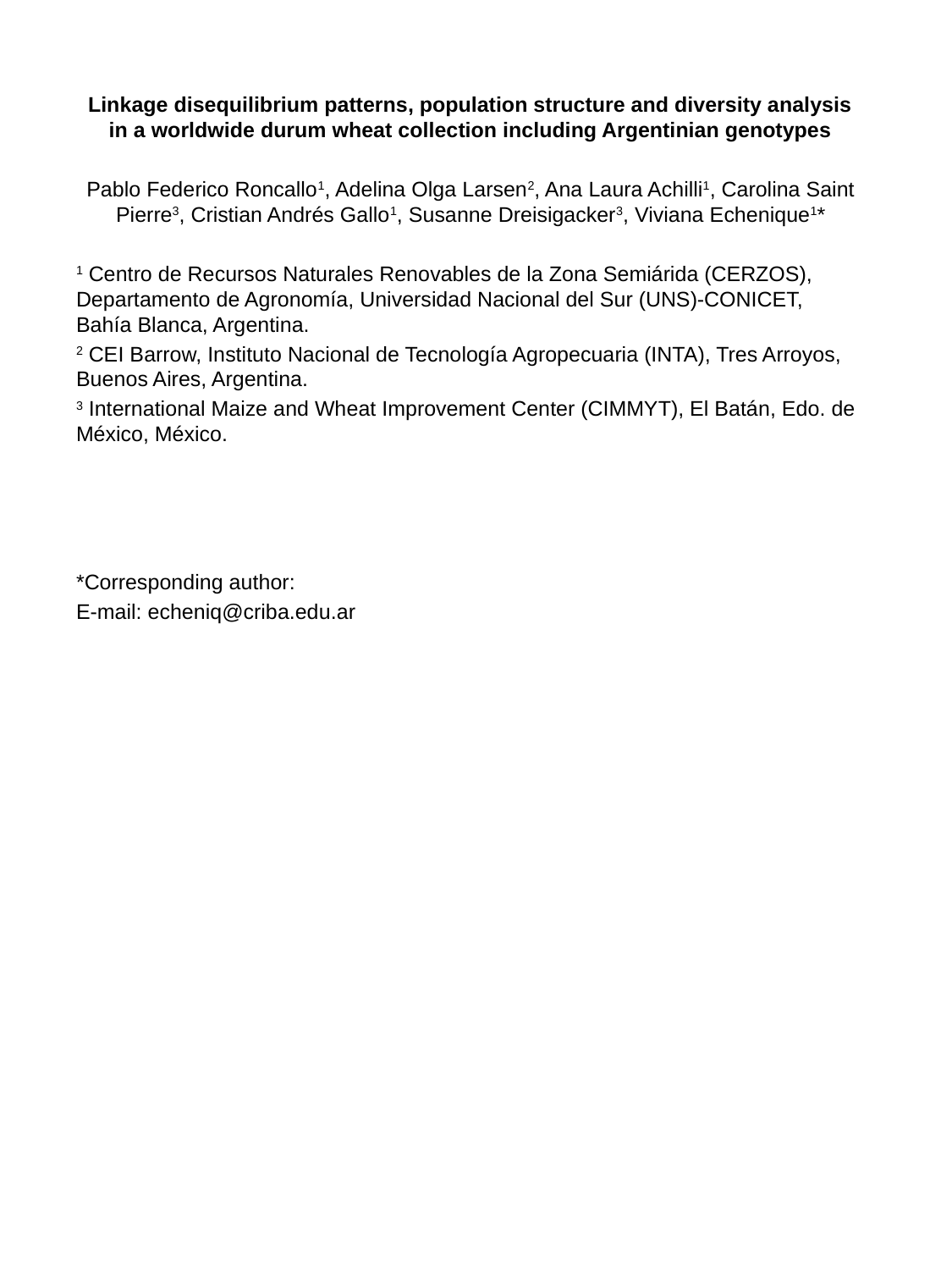

Linkage disequilibrium patterns, population structure and diversity analysis in a worldwide durum wheat collection including Argentinian genotypes
Pablo Federico Roncallo1, Adelina Olga Larsen2, Ana Laura Achilli1, Carolina Saint Pierre3, Cristian Andrés Gallo1, Susanne Dreisigacker3, Viviana Echenique1*
1 Centro de Recursos Naturales Renovables de la Zona Semiárida (CERZOS), Departamento de Agronomía, Universidad Nacional del Sur (UNS)-CONICET, Bahía Blanca, Argentina.
2 CEI Barrow, Instituto Nacional de Tecnología Agropecuaria (INTA), Tres Arroyos, Buenos Aires, Argentina.
3 International Maize and Wheat Improvement Center (CIMMYT), El Batán, Edo. de México, México.
*Corresponding author:
E-mail: echeniq@criba.edu.ar

## Slide 2
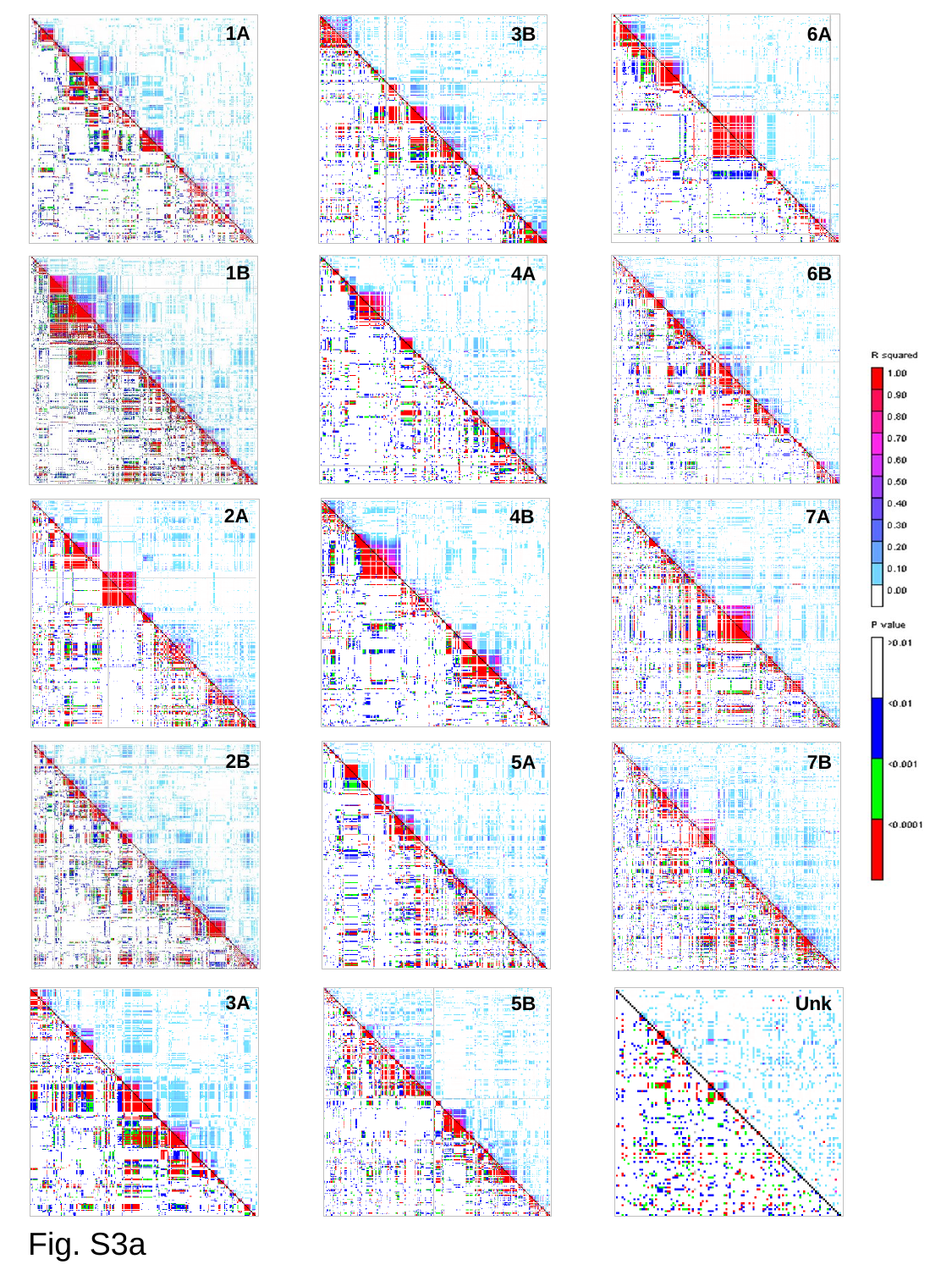

1A
3B
6A
1B
4A
6B
2A
4B
7A
2B
5A
7B
3A
5B
Unk
Fig. S3a

## Slide 3
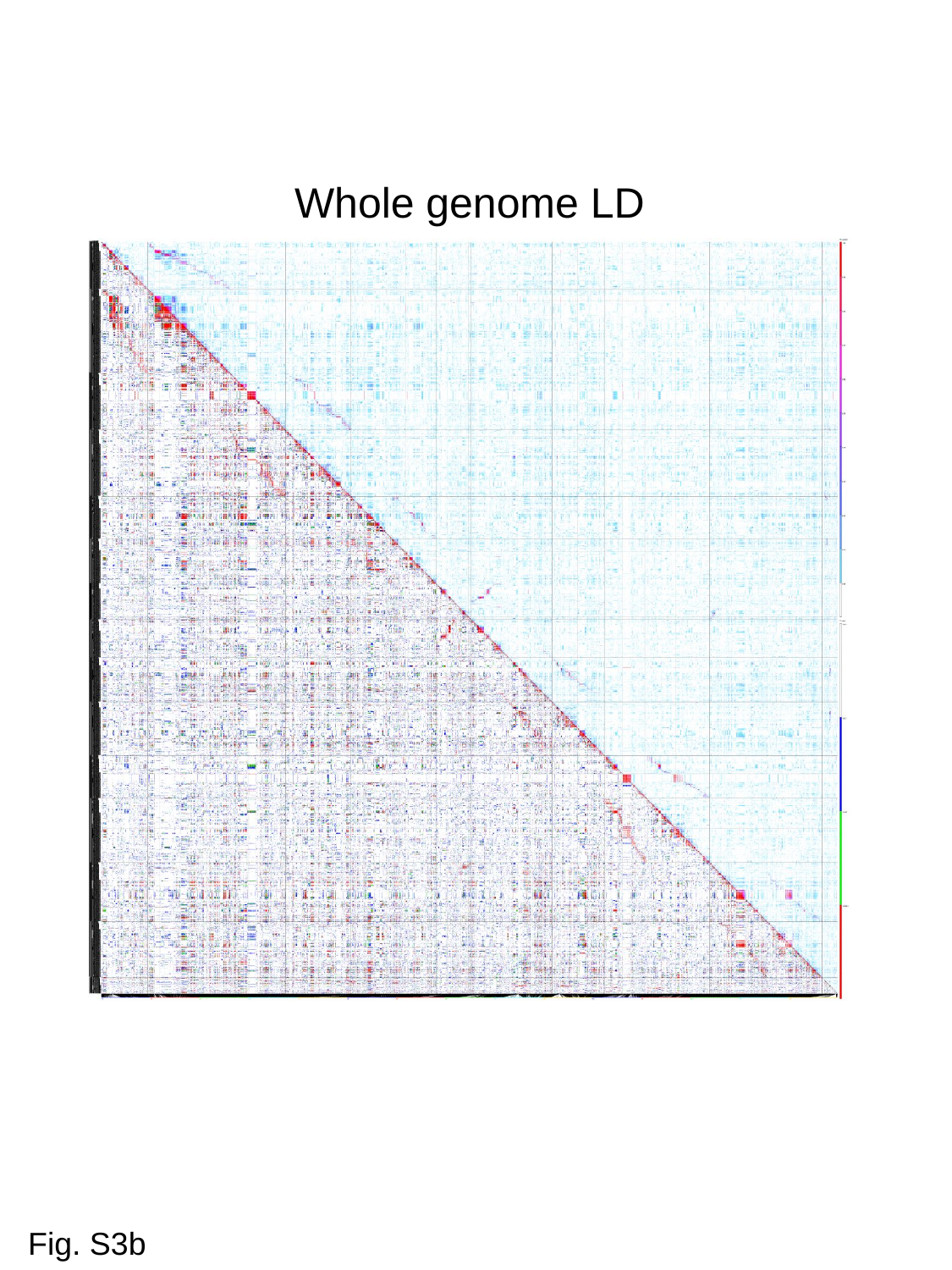

Whole genome LD
Fig. S3b
