# Supplementary material for: Linkage disequilibrium patterns, population structure and diversity analysis in a worldwide durum wheat collection including Argentinian genotypes
Source: BMC Genomics. 2021 Apr 5;22:233. doi: 10.1186/s12864-021-07519-z (PMC8022437; doi:10.1186/s12864-021-07519-z)
Supplement: Supplementary file 9 — Additional file 9. [file 12864_2021_7519_MOESM9_ESM.pptx]

## Slide 1
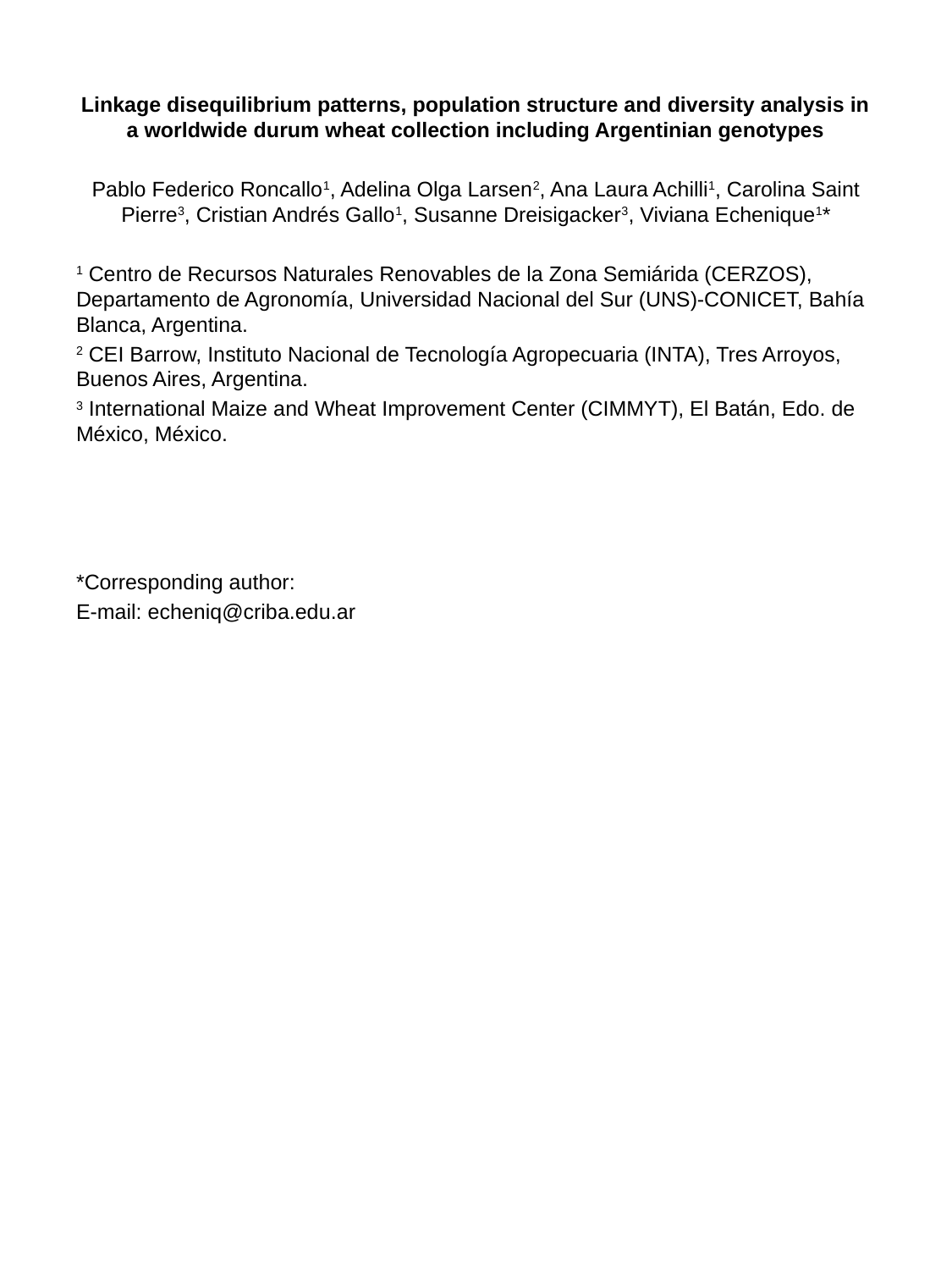

Linkage disequilibrium patterns, population structure and diversity analysis in a worldwide durum wheat collection including Argentinian genotypes
Pablo Federico Roncallo1, Adelina Olga Larsen2, Ana Laura Achilli1, Carolina Saint Pierre3, Cristian Andrés Gallo1, Susanne Dreisigacker3, Viviana Echenique1*
1 Centro de Recursos Naturales Renovables de la Zona Semiárida (CERZOS), Departamento de Agronomía, Universidad Nacional del Sur (UNS)-CONICET, Bahía Blanca, Argentina.
2 CEI Barrow, Instituto Nacional de Tecnología Agropecuaria (INTA), Tres Arroyos, Buenos Aires, Argentina.
3 International Maize and Wheat Improvement Center (CIMMYT), El Batán, Edo. de México, México.
*Corresponding author:
E-mail: echeniq@criba.edu.ar

## Slide 2
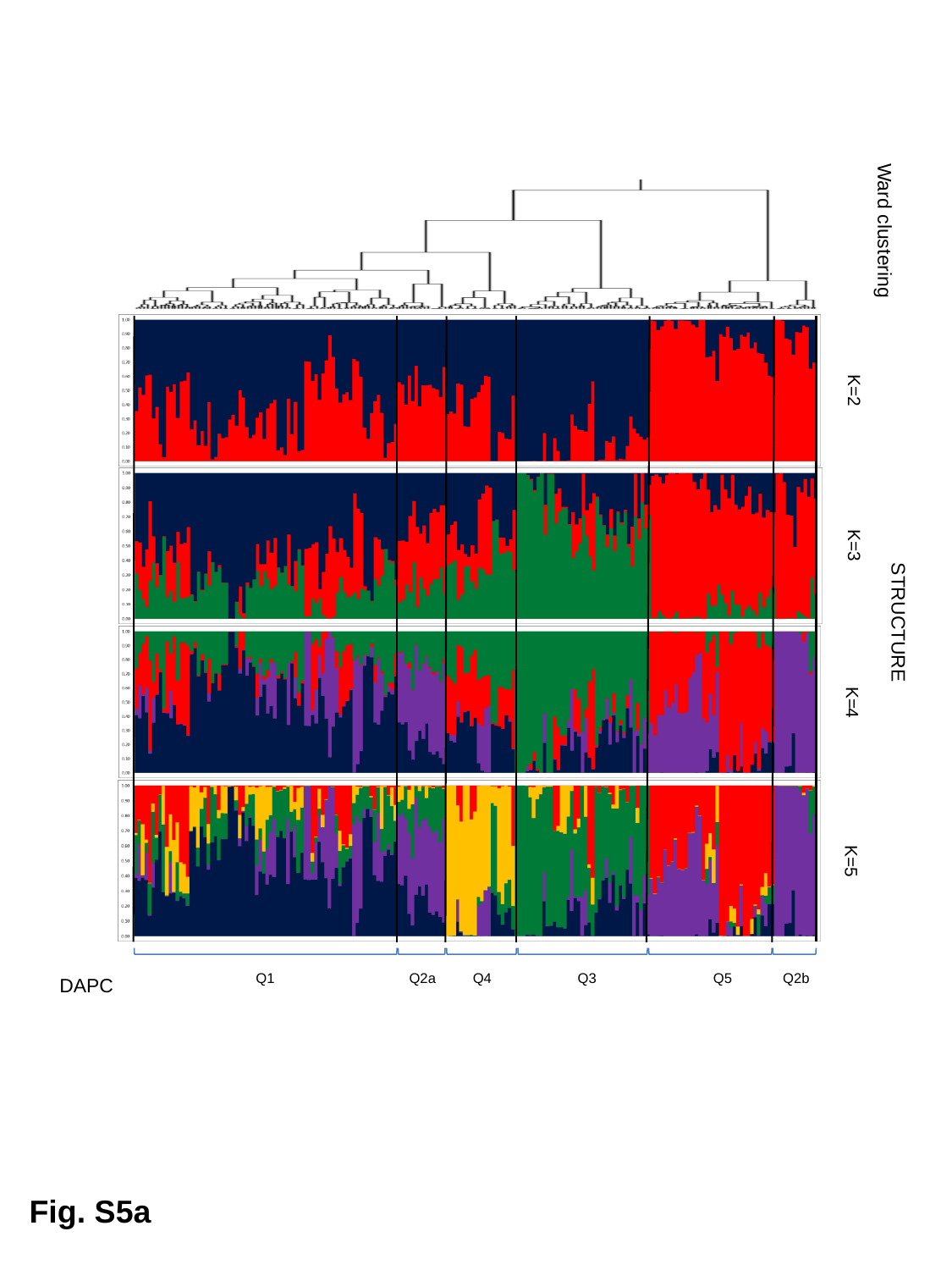

Ward clustering
K=2
K=3
STRUCTURE
K=4
K=5
Q1
Q2b
Q2a
Q4
Q3
Q5
DAPC
Fig. S5a

## Slide 3
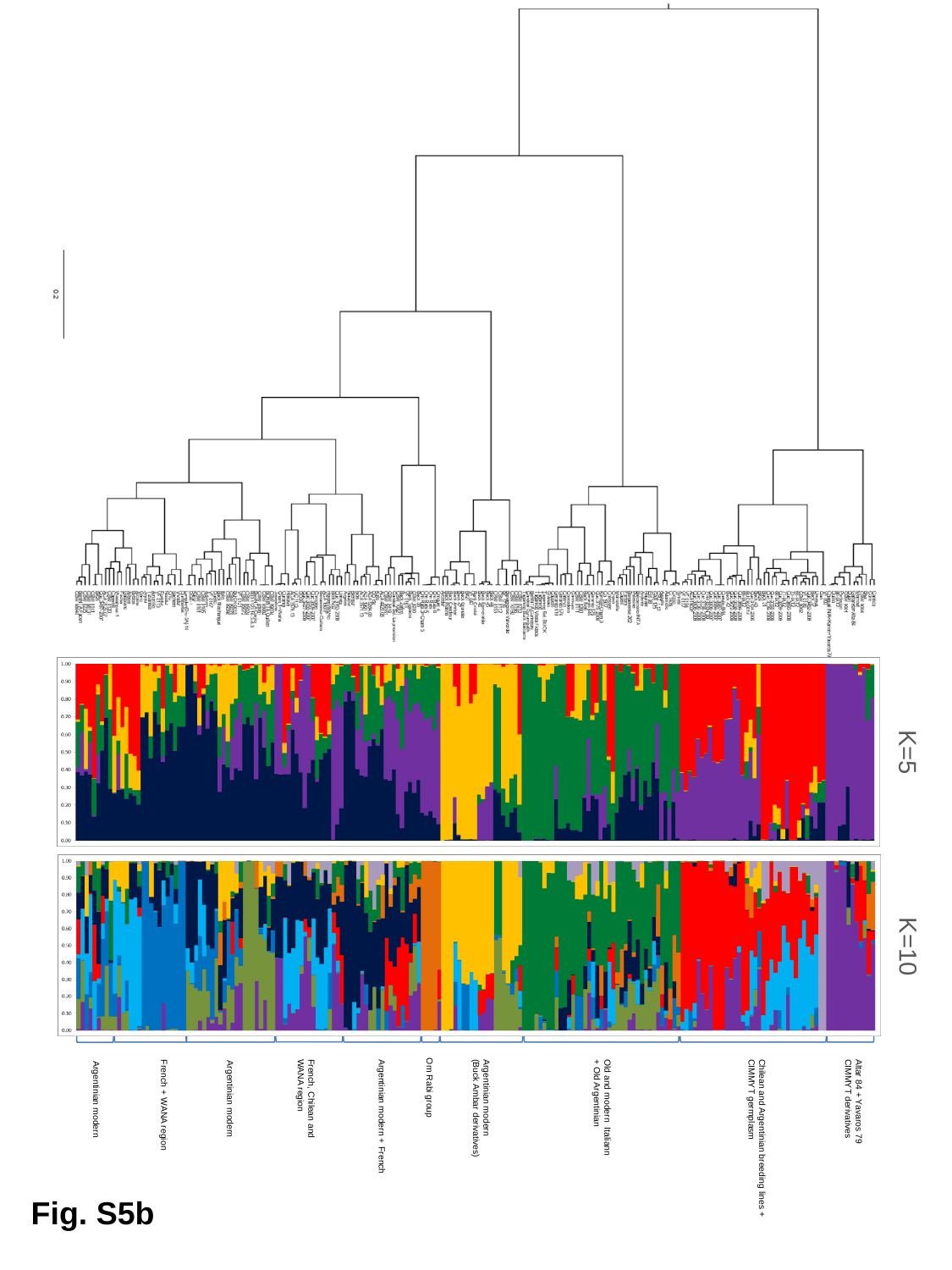

K=5
K=10
Old and modern Italiann
+ Old Argentinian
Altar 84 + Yavaros 79
CIMMYT derivatives
French, Chilean and
WANA region
Chilean and Argentinian breeding lines +
CIMMYT germplasm
Argentinian modern
(Buck Ambar derivatives)
Om Rabi group
Argentinian modern + French
French + WANA region
Argentinian modern
Argentinian modern
Fig. S5b
